# Thyroid function and epilepsy: a two-sample Mendelian randomization study

**DOI:** 10.3389/fnhum.2023.1295749

**Published:** 2024-01-17

**Authors:** Di Lu, Yunming Wang, Yanfeng Yang, Huaqiang Zhang, Xiaotong Fan, Sichang Chen, Penghu Wei, Yongzhi Shan, Guoguang Zhao

**Affiliations:** ^1^Department of Neurosurgery, Xuanwu Hospital, Capital Medical University, Beijing, China; ^2^Clinical Research Center for Epilepsy, Capital Medical University, Beijing, China; ^3^Beijing Municipal Geriatric Medical Research Center, Beijing, China

**Keywords:** thyroid, epilepsy, Mendelian randomization, hippocampal sclerosis, juvenile absence epilepsy, childhood absence epilepsy

## Abstract

**Background:**

Thyroid hormones (THs) play a crucial role in regulating various biological processes, particularly the normal development and functioning of the central nervous system (CNS). Epilepsy is a prevalent neurological disorder with multiple etiologies. Further in-depth research on the role of thyroid hormones in epilepsy is warranted.

**Methods:**

Genome-wide association study (GWAS) data for thyroid function and epilepsy were obtained from the ThyroidOmics Consortium and the International League Against Epilepsy (ILAE) Consortium cohort, respectively. A total of five indicators of thyroid function and ten types of epilepsy were included in the analysis. Two-sample Mendelian randomization (MR) analyses were conducted to investigate potential causal relations between thyroid functions and various epilepsies. Multiple testing correction was performed using Bonferroni correction. Heterogeneity was calculated with the Cochran’s Q statistic test. Horizontal pleiotropy was evaluated by the MR-Egger regression intercept. The sensitivity was also examined by leave-one-out strategy.

**Results:**

The findings indicated the absence of any causal relationship between abnormalities in thyroid hormone and various types of epilepsy. The study analyzed the odds ratio (OR) between thyroid hormones and various types of epilepsy in five scenarios, including free thyroxine (FT4) on focal epilepsy with hippocampal sclerosis (IVW, OR = 0.9838, *p* = 0.02223), hyperthyroidism on juvenile absence epilepsy (IVW, OR = 0.9952, *p* = 0.03777), hypothyroidism on focal epilepsy with hippocampal sclerosis (IVW, OR = 1.0075, *p* = 0.01951), autoimmune thyroid diseases (AITDs) on generalized epilepsy in all documented cases (weighted mode, OR = 1.0846, *p* = 0.0346) and on childhood absence epilepsy (IVW, OR = 1.0050, *p* = 0.04555). After Bonferroni correction, none of the above results showed statistically significant differences.

**Conclusion:**

This study indicates that there is no causal relationship between thyroid-related disorders and various types of epilepsy. Future research should aim to avoid potential confounding factors that might impact the study.

## 1 Introduction

Thyroid hormones (THs), including triiodothyronine (T3) and thyroxine (T4), are iodinated derivatives of tyrosine that are secreted by the thyroid gland and play a vital role in regulating diverse biological processes *in vivo*, such as growth, development, differentiation, and metabolism (Taylor and Ritchie, 2007). Normal thyroid function and appropriate TH levels are essential for proper development and organ function, particularly in the central nervous system (CNS) (Prezioso et al., 2018). Thyroid hormone disorders can result in decreased inhibitory neurons and ion deposition and ultimately culminate in neuronal death and apoptosis (Sawicka-Gutaj et al., 2022).

Epilepsy is a common neurological disorder characterized by transient or permanent neuronal damage caused by repetitive, spontaneous synchronous abnormal discharges of neurons. It is an intractable disease with various heterogeneities in clinical manifestations, mainly including spontaneous convulsions with or without a loss of consciousness (Thijs et al., 2019). The etiology and pathogenesis of epilepsy are very complex, including genetic, structural, metabolic, immune, infectious, and unknown factors (Scheffer et al., 2017). As metabolic disorders that may affect neurological functions, thyroid hormone abnormalities have gradually attracted increasing attention in epilepsy research. However, the causal direction between thyroid dysfunction and epilepsies remains unknown.

Mendelian randomization (MR) is an emerging approach for exploring potential causality with the development of large-scale GWASs. MR draws on the ideas of Mendel’s laws of genetic inheritance, including the law of segregation and the law of independent assortment (Sanderson et al., 2022). Using the genetic variants as bridges, this method can construct the potentially causal relationship between variables. It avoids the drawbacks of the conventional approaches, in which correlation does not mean causation. This study aims to explore the potential causal relationship between thyroid dysfunction and epilepsy using the Mendelian randomization method.

## 2 Materials and methods

### 2.1 Ethics statement

There are no ethics concerns involved with the study, and all data were acquired from publicly available datasets.

### 2.2 Study design

**FIGURE 1:**
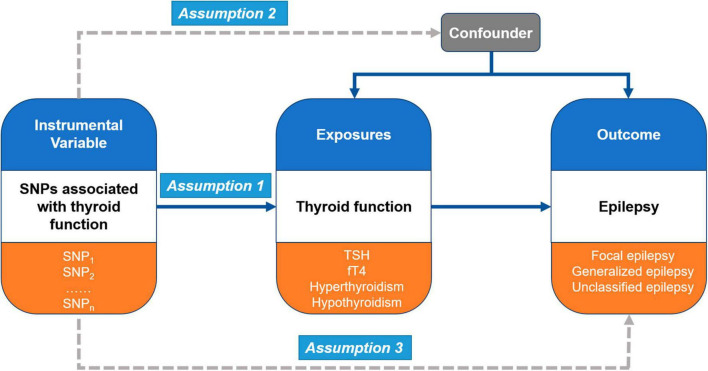
Overall design of Mendelian randomization (MR) analysis in this study.

In the present study, Mendelian randomization (MR) analysis was performed to investigate causal pathways between thyroid function and epilepsy (Figure 1). MR analysis is a statistical approach that uses instrumental variables (IVs) to test causal relationships between exposures and outcomes (Smith and Ebrahim, 2003). It is based on three main assumptions. First, the genetic variants (multiple single-nucleotide polymorphisms, SNPs) recognized as IVs should be robustly associated with the exposure (thyroid function) (Assumption 1). Second, these genetic variants should not be associated with any confounders between the exposure and outcomes (different types of epilepsy) (Assumption 2). Third, the genetic variants as IVs of an exposure should affect the risk of the outcome merely through the exposure, without any other alternative pathways (Assumption 3).

### 2.3 Data sources

#### 2.3.1 GWAS data for thyroid function

We obtained the genome-wide association study (GWAS) summary statistics of thyroid function from the ThyroidOmics Consortium.^1^ The main study population is of European ancestry. It is a large meta-analysis of 47 GWASs. The main indicators of thyroid function in ThyroidOmics include thyroid-stimulating hormone (TSH) (22 independent cohorts with 54,288 subjects), free thyroxine (FT4) (19 cohorts with 49,269 subjects), hyperthyroidism (1,840 cases and 49,983 controls), and hypothyroidism (3,440 cases and 49,983 controls) (Teumer et al., 2018). In addition, GWASs of autoimmune thyroid diseases (AITDs) were enrolled in this study, including Graves’ disease (2,400 cases), Hashimoto’s thyroiditis (397 cases), other nonautoimmune hypothyroidism (27,437 cases), and healthy controls (755,172 subjects) (Kjaergaard et al., 2022).

#### 2.3.2 GWAS data for epilepsy

We obtained GWAS summary statistics data in patients with epilepsy from the International League Against Epilepsy (ILAE) Consortium cohort.^2^ The primary data consisted of the following types of epilepsy: focal epilepsy, documented hippocampal sclerosis (803 cases); focal epilepsy, all documented cases (9,671 cases); focal epilepsy, documented lesions other than hippocampal sclerosis (3,070 cases); juvenile absence epilepsy (415 cases); generalized epilepsy with tonic-clonic seizures (228 cases); juvenile myoclonic epilepsy (1,181 cases); focal epilepsy, documented lesion negative (2,716 cases); epilepsy, all documented cases (15,212 cases); generalized epilepsy, all documented cases (3,769 cases); and childhood absence epilepsy (793 cases). It also included data from 29,677 healthy controls. The sample population was mainly of European ancestry. In addition, we obtained several other GWAS datasets from MR-base^3^ to further verify the results.

### 2.4 Instrumental variable selection

The strength of IVs was checked by means of the F-statistic. F-statistics > 10 were considered robust IVs. The genome-wide significance level was set at *p* < 5 × 10^–8^. To avoid a specific exposure SNP that was not present in the outcome, a proxy SNP was selected. The minimum linkage disequilibrium (LD) for the *r*^2^ value was set at 0.8. SNPs were discarded if no suitable proxy was available. This study allowed palindromic SNPs, and the minor allele frequency (MAF) threshold for aligning palindromes was set at 0.3. According to the ThyroidOmics Consortium database, we recognized 60 SNPs associated with TSH, 30 SNPs associated with FT4, 7 SNPs associated with hyperthyroidism, and 18 SNPs associated with hypothyroidism. We also identified a total of 86 SNPs related to AITDs (Supplementary Table 1). To avoid confounding factors, we then checked the potential phenotypes associated with the chosen SNPs using the GWAS Catalog,^4^ PhenoScanner,^5^ and PubMed.^6^

### 2.5 MR analysis

We then performed two-sample MR analysis using the TwoSampleMR package (version: 0.5.5) in R. The inverse variance-weighted (IVW) method was used as the main analysis method. It uses the inverse variance weights to calculate the weighted mean to assess the causality of the study (Burgess et al., 2020). In addition, other methods, including Wald ratio, MR-Egger, weighted median, and weighted mode, were also employed. Wald ratio method was performed when only one IV was obtained (Qin et al., 2023). The MR-Egger method could be used to assess potential pleiotropy by evaluating the intercept (Ren et al., 2023). The weighted median was performed when more than 50% of the weight is derived from valid IVs (Luo et al., 2022). If the relationship between thyroid function and epilepsy is observed consistently across multiple methods, we can have greater confidence in the results, as these methods have various assumptions regarding the validity of the IVs (Slaney et al., 2023). We then used Bonferroni correction to adjust the alpha (α) level for multiple hypothesis tests in order to control the probability of committing a type I error. The modified thresholds were determined based on the number of methods used in each analysis (Chong et al., 2023). For a single IV with Wald ratio method, no correction of the *p*-value is necessary. In this study, the corrected α level was 0.0125 (0.05/4). A *p*-value less than 0.0125 was considered statistically significant. A *p*-value greater than 0.0125 was considered as non-significant.

### 2.6 Sensitivity analyses

To validate the robustness of the above MR results, a series of sensitivity analyses were performed, including a heterogeneity test, pleiotropy test, and leave-one-out sensitivity test.

#### 2.6.1 Heterogeneity test

The heterogeneity among instrumental variables may result from differences in populations and studies. Cochran’s Q statistic was calculated in the IVW and MR-Egger methods to test the heterogeneity between each IV. A Q_pval greater than 0.05 indicated an absence of heterogeneity.

#### 2.6.2 Pleiotropy test

Horizontal pleiotropy indicates that the IVs influence the outcome through a pathway other than the exposure, which violates Assumption 3. The Egger regression intercept was calculated to assess horizontal pleiotropy, and a funnel plot was used to visualize the results.

#### 2.6.3 Leave-one-out sensitivity test

The leave-one-out sensitivity test was conducted to detect the effect of a single IV on the MR results. Specifically, this approach can identify potential single IVs that may significantly affect the results by eliminating each IV one by one and recalculating the MR results.

### 2.7 Mathematical formulas

Beta and odds ratio (OR) are important parameters in MR analysis. The OR value allows more intuitive interpretation of the results. ORs greater than 1 indicate increased risk of outcomes, while ORs less than 1 indicate decreased risk. Therefore, for results with a *p*-value < 0.05 before the Bonferroni correction, we used the following formulas to convert β to OR and the results were visualized with RStudio.


95%⁢C⁢I=b⁢e⁢t⁢a±1.96⁢s⁢e

O⁢R=E⁢X⁢P⁢(b⁢e⁢t⁢a)

O⁢R⁢_⁢95%⁢C⁢I=E⁢X⁢P⁢(b⁢e⁢t⁢a±1.96⁢s⁢e)

## 3 Results

**FIGURE 2:**
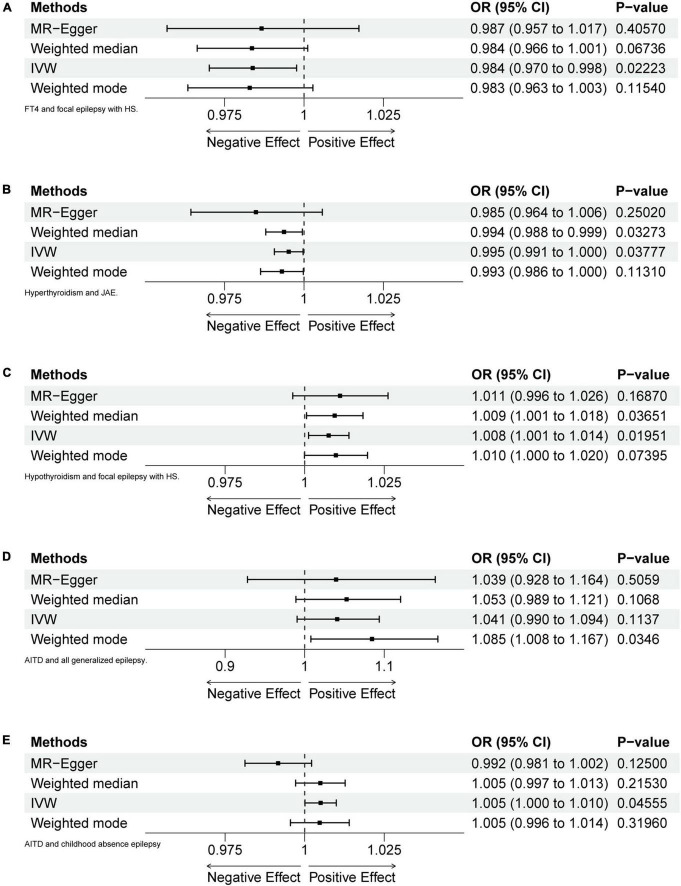
Forest plots of MR analyses. **(A)** FT4 and focal epilepsy with hippocampal sclerosis (HS). **(B)** Hyperthyroidism and juvenile absence epilepsy (JAE). **(C)** Hypothyroidism and focal epilepsy with HS. **(D)** Autoimmune thyroid diseases (AITD) and generalized epilepsy (all documented cases). **(E)** AITD and childhood absence epilepsy (CAE).

In this study, 50 potential relationships were examined (5 thyroid function indicators × 10 types of epilepsy) (Supplementary Table 1). Further calculations of OR were performed for five scenarios between thyroid function and various types of epilepsy (Figure 2). We validated the Assumption 1 of MR analysis by manually searching the GWAS Catalog, PhenoScanner websites, and PubMed. All IVs were robustly associated with the thyroid function (exposures). The similarity in the population composition (Europeans) between the exposures and outcomes ensured the validity of Assumption 2.

### 3.1 Relationship between FT4 and epilepsy

**FIGURE 3:**
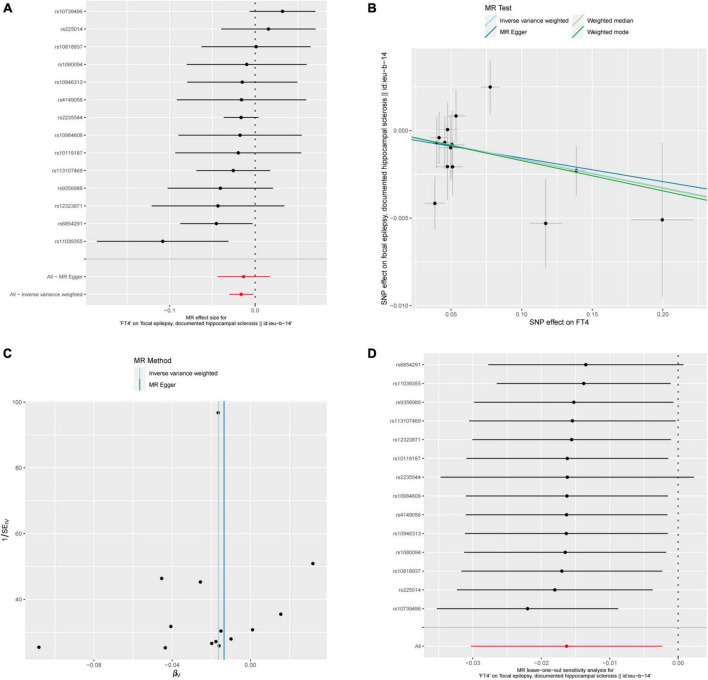
FT4 and focal epilepsy with hippocampal sclerosis. **(A)** Forest plot of single-SNP MR. **(B)** Comparison of results using different MR methods. **(C)** Funnel plot. **(D)** Leave-one-out sensitivity analysis.

After calculation, 14 out of 30 SNPs associated with FT4 were used for MR analysis (Figure 3). Through comprehensive MR analysis, we did not identify a causal relationship between FT4 and any form of epilepsy. Regarding FT4 and focal epilepsy with hippocampal sclerosis, the IVW method yielded an OR of 0.9838, with a 95% confidence interval of 0.9702–0.9977 and a *p*-value of 0.02223. MR-Egger, weighted median, and weighted mode yielded similar results, with ORs of 0.9866, 0.9836, and 0.9829, respectively (Figure 2A). Nevertheless, upon applying Bonferroni correction, no statistically significant causal relationships were identified (0.02223 > 0.0125). In heterogeneity statistics, the Q_pval was 0.1865 in the MR-Egger method and 0.2405 in the IVW method, which indicated that there was no significant heterogeneity in this result, as both values were greater than 0.05. The Egger regression intercept was −0.00023, and the directionality *p*-value was 0.84, indicating that no evidence for horizontal pleiotropy was observed. This result aligns with the Assumption 3. Further investigation showed that there was also no obvious causal relationship between FT4 level and other types of epilepsy, as all *p*-values of different MR methods were greater than 0.05. Detailed results are shown in Supplementary Table 1.

### 3.2 Relationship between TSH and epilepsy

Forty-eight out of 60 SNPs associated with TSH were analyzed with different types of epilepsy. However, our study did not find any evidence of a possible causal relationship, as all *p*-values of different MR methods were greater than 0.05. Detailed results are shown in Supplementary Table 1.

### 3.3 Relationship between hyperthyroidism and epilepsy

**FIGURE 4:**
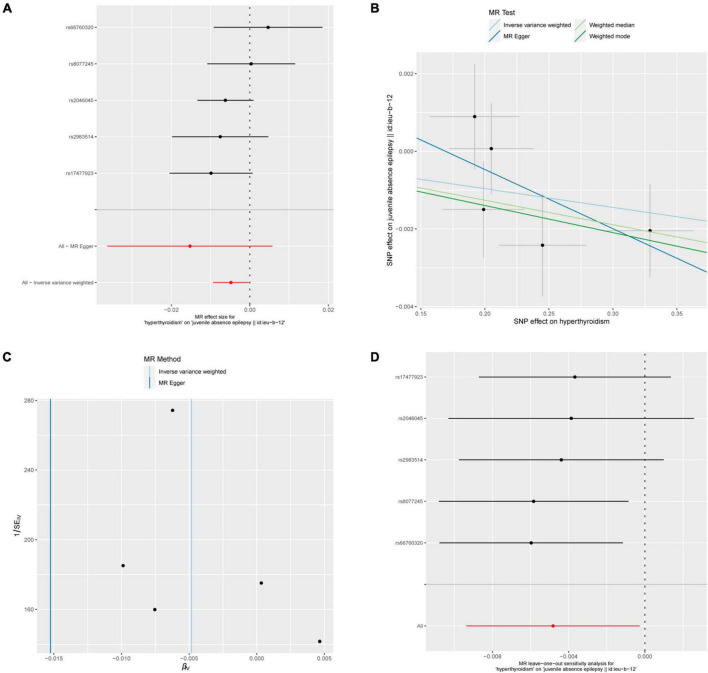
Hyperthyroidism and juvenile absence epilepsy. **(A)** Forest plot of single-SNP MR. **(B)** Comparison of results using different MR methods. **(C)** Funnel plot. **(D)** Leave-one-out sensitivity analysis.

Five out of 7 SNPs associated with hyperthyroidism were analyzed with different types of epilepsy (Figure 4). No causal relationship between hyperthyroidism and any form of epilepsy was found through various MR analyses. Taking hyperthyroidism and juvenile absence epilepsy (JAE) as examples, the OR obtained through the IVW method was 0.9952, with a 95% confidence interval of 0.9907–0.9997 and a *p*-value of 0.03777. Using the weighted median method, the OR was 0.9937, with a 95% confidence interval of 0.9880–0.9995 and a *p*-value of 0.03273. MR-Egger and weighted mode yielded similar results, with ORs of 0.9849 and 0.9930, respectively (Figure 2B). However, when employing Bonferroni correction to control for multiple comparisons, no potential causal relationships emerged.

The heterogeneity statistics yielded Q_pval values of 0.4169 in the MR-Egger method and 0.4291 in the IVW method. Given that both values were greater than 0.05, we concluded that there was no significant heterogeneity in the results. Additionally, the Egger regression intercept was 0.0026, and the directionality *p*-value was 0.393, indicating no evidence of horizontal pleiotropy. Further investigation showed that there was no obvious causal relationship between hyperthyroidism and other types of epilepsy, as all *p*-values of the different MR methods were greater than 0.05. Detailed results are shown in Supplementary Table 1.

### 3.4 Relationship between hypothyroidism and epilepsy

**FIGURE 5:**
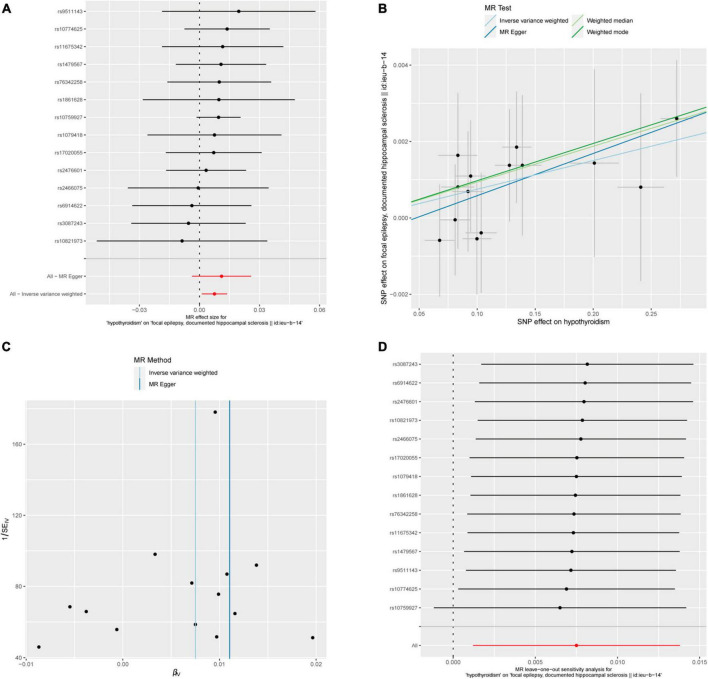
Hypothyroidism and focal epilepsy with hippocampal sclerosis. **(A)** Forest plot of single-SNP MR. **(B)** Comparison of results using different MR methods. **(C)** Funnel plot. **(D)** Leave-one-out sensitivity analysis.

Fourteen out of 18 SNPs associated with hypothyroidism were analyzed with different types of epilepsy (Figure 5). The MR analysis did not reveal a causal relationship between hypothyroidism and any type of epilepsy. When analyzing the relationship between hypothyroidism and focal epilepsy with hippocampal sclerosis, the results from the IVW method showed an OR of 1.0075, with a 95% confidence interval of 1.0012–1.0139 and a *p*-value of 0.01951. The results from the weighted median method were as follows: OR = 1.0094, 95% confidence interval = 1.0006–1.0183, *p* = 0.03651. MR-Egger and weighted mode yielded similar results, with ORs of 1.0111 and 1.0098, respectively (Figure 2C). It can be observed that, although the OR confidence interval does not include 1, the results of the OR, due to their proximity to 1, cannot conclusively establish the presence of a causal relationship. Bonferroni correction further confirmed that the *p*-values did not reach the statistical significance level for establishing a causal relationship.

The heterogeneity statistics using the MR-Egger method and IVW approach yielded Q_pval values of 0.9951 and 0.9965, respectively, indicating the absence of significant heterogeneity in this finding, as both values were above the threshold of 0.05. Furthermore, the Egger regression intercept was calculated as −0.00052, and the directionality *p*-value was 0.613, suggesting no evidence of horizontal pleiotropy in the data. Subsequent examination revealed that a discernible causal association between hyperthyroidism and other forms of epilepsy was not evident, as indicated by the fact that all *p*-values obtained from various MR methods were above the threshold of 0.05. Detailed results are shown in Supplementary Table 1.

### 3.5 Relationship between AITDs and epilepsy

**FIGURE 6:**
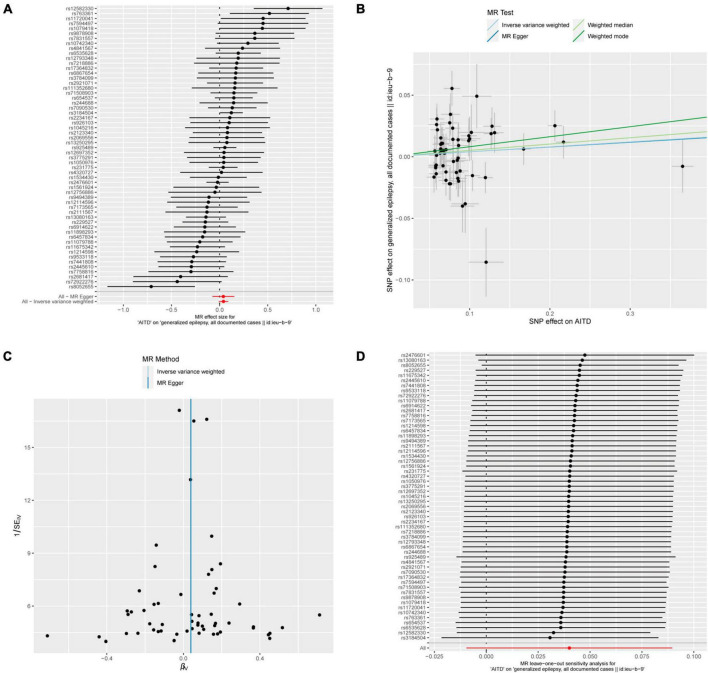
AITDs and generalized epilepsy, all documented cases. **(A)** Forest plot of single-SNP MR. **(B)** Comparison of results using different MR methods. **(C)** Funnel plot. **(D)** Leave-one-out sensitivity analysis.

**FIGURE 7:**
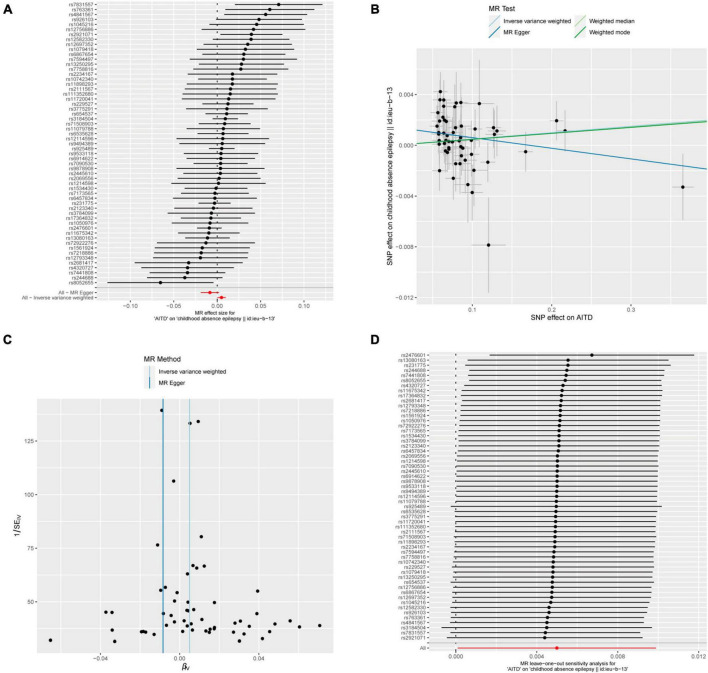
AITDs and childhood absence epilepsy. **(A)** Forest plot of single-SNP MR. **(B)** Comparison of results using different MR methods. **(C)** Funnel plot. **(D)** Leave-one-out sensitivity analysis.

Fifty-seven out of 86 SNPs associated with AITDs were analyzed with different types of epilepsy (Figures 6, 7). The conducted MR studies did not find a causal relationship between AITDs and various forms of epilepsy. Regarding AITDs and generalized epilepsy (all documented cases), using the weighted mode method yielded an OR of 1.0846, with a 95% confidence interval of 1.0077–1.1673, and a *p*-value of 0.0346. Nonetheless, it was found that the statistical significance of the causal relationship was not maintained after applying the Bonferroni correction. The statistical methods of IVW, MR-Egger, and weighted median produced comparable outcomes, with ORs of 1.0408, 1.0394, and 1.0527, respectively (Figure 2D). The calculated OR values obtained from these three alternative methods were all close to 1 and the *p*-values were all greater than 0.05, indicating no causal relationship.

In addition, in the context of heterogeneity statistics, the Q_pval was 0.000439 in the MR-Egger method and 0.0005967 in the IVW method. The possibility of heterogeneity in this outcome was taken into consideration because both values were below the significance threshold of 0.05. The Egger regression intercept was 0.00014, and the directionality *p*-value was 0.98, suggesting no evidence of horizontal pleiotropy.

Meanwhile, the results of the causal relationship study between AITDs and childhood absence epilepsy (CAE) were particularly noteworthy. MR analysis indicates that the OR obtained through the IVW method is 1.0050, with a 95% confidence interval of 1.0001–1.0099, and a *p*-value of 0.04555. After applying Bonferroni correction, the *p*-value could not establish statistical significance for a causal relationship. Weighted median and weighted mode yielded similar results, with ORs of 1.0049 and 1.0047, respectively (Figure 2E). However, we obtained the opposite findings in MR-Egger, with an OR of 0.9916 (less than 1). Although the *p*-value for this method was 0.125, indicating a lack of statistical significance, this still diminished the robustness and reliability of the results obtained by the other three methods.

Regarding the heterogeneity statistics, the Q_pval was 0.513 in the MR-Egger method and 0.2803 in the IVW method. Both values were greater than 0.05, suggesting no significant heterogeneity in these results. However, it is worth noting that the Egger regression intercept was 0.0014, and the directionality *p*-value was 0.00759, which indicated that there may be horizontal pleiotropy in the result. Upon further investigation, no obvious causal relationship was found between AITDs and other types of epilepsy, as all *p*-values from different MR methods were greater than 0.05. Detailed results are shown in Supplementary Table 1.

## 4 Discussion

Thyroid hormones (THs) are essential for neurodevelopment. During the fetal and neonatal periods, THs can influence neuronal proliferation, migration, synaptogenesis, myelination, and dendritic cell differentiation (Remaud et al., 2014). Both hyperthyroidism and hypothyroidism have the potential to disrupt mitochondrial function through genetic and nongenetic pathways, leading to oxidative/antioxidative imbalance (oxidative stress) and the generation of reactive oxygen species (ROS) (Maity et al., 2013; Peixoto et al., 2021). As a common and challenging neurological disorder, the etiology of epilepsy is heterogenous and gaining widespread attention. A substantial portion of epilepsy can be attributable to genetic factors (Thomas and Berkovic, 2014). The occurrence of some types of epilepsy is associated with neurodevelopmental factors, such as gray matter heterotopia, focal cortical dysplasia, and hypothalamic hamartoma. Additionally, certain anti-epileptic drugs may affect the function of thyroid hormones. Currently, there is limited research on the potential relationship between thyroid hormone abnormalities and epilepsy. The aim of this study was to determine whether there is a causal relationship between these two types of diseases.

Mendelian randomization is a novel research method that utilizes SNP data from large population samples to directly assess causal relationships between exposures and outcome factors. This study employed Mendelian randomization to investigate the relationship between thyroid hormones and epilepsy using genetic variants with minimal disturbance factors. The results showed that there was no statistically significant causal relationship between thyroid hormone abnormalities and various types of epilepsy. We also conducted a focused analysis of the OR values for exposure and outcome factors, including hypothyroidism, FT4 levels, in focal epilepsy with hippocampal sclerosis, hyperthyroidism in JAE, and AITD in CAE and in generalized epilepsy (all documented cases).

The thyroid gland, a crucial endocrine organ in humans, is the first endocrine organ to form during fetal development (Yoon et al., 2023). Subclinical and overt thyroid dysfunction are common diseases of the endocrine system. The incidence rates vary across different regions, with the coastal population having a higher risk of developing thyroid abnormalities compared to inland areas (Wang et al., 2021). Genetic susceptibility is closely associated with the onset of thyroid diseases, such as autoimmune thyroid diseases (AITD) (Medici et al., 2014). Thyroid dysfunction is primarily attributed to thyroid autoimmunity in iodine-replete areas. In addition, iodine, a significant environmental factor, plays a critical role in the risk of thyroid disease. Nodular thyroid disorders are more frequently observed in iodine-deficient areas (Taylor et al., 2018). Nevertheless, various factors, including smoking, aging, ethnicity, and medication also contribute to the epidemiology of thyroid disease (Taylor et al., 2018). Anti-epileptic drugs, such as valproate and carbamazepine, can also impact thyroid function (Rochtus et al., 2022). Additionally, environmental factors can increase the risk of diseases like AITD by influencing epigenetic modifications of genes (Tomer, 2014). Multiple etiological factors contribute to the complexity of thyroid disease in this study, which may be a significant factor contributing to the lack of a clear relationship between the thyroid and epilepsy.

According to the latest guidelines provided by the International League Against Epilepsy (ILAE), the diagnosis of epilepsy should be divided into three levels, seizure type, epilepsy type, and epilepsy syndrome, which can be broadly classified into the following categories: generalized epilepsy syndromes, focal epilepsy syndromes, focal and generalized epilepsy syndromes, unknown, and epilepsy syndromes associated with developmental epileptic encephalopathy (DEE) or progressive neurological deterioration (Wirrell et al., 2022). An etiologic diagnosis should be considered at each step along the diagnostic pathway. Regardless of the origins of epileptic activity, a patient’s epilepsy may be classified into more than one etiology (Scheffer et al., 2017). Given the results of the present study, we focused on a structural etiology called hippocampal sclerosis (HS), which is frequently recognized in mesial temporal lobe epilepsy (mTLE) in focal epilepsy, and other generalized epilepsy syndromes, including CAE and JAE, which are attributed to more than one etiology.

Temporal lobe epilepsy, especially mTLE, is the most common type of epilepsy (Engel, 2001). The primary pathological entity in mTLE is HS. Microscopically, varying degrees of neuronal loss and gliosis can be observed in distinct subregions of the hippocampus, such as CA1-CA4, the dentate gyrus, and the subiculum (Thom, 2014). However, the exact mechanisms underlying HS remain unclear. Previous studies proposed a combination of multiple factors, including increased genetic susceptibility and acquired neural injuries, such as febrile seizures and encephalitis (Kuks et al., 1993; Popkirov et al., 2017). In addition, TH signaling plays a cell-autonomous role in regulating hippocampal neurogenesis in adults (Mayerl et al., 2020). Genomics and cerebrospinal fluid (CSF) analyses have provided evidence supporting the potential relationship between TH perturbation and HS (Nelson et al., 2016). In addition to epilepsy, HS is also significantly associated with dementia in elderly individuals. Trieu et al. (2018) demonstrated that high levels of TSH and thyroid antibodies were associated with HS. In non-Alzheimer’s neurodegeneration, two risk alleles (rs704180 and rs73069071) on chromosome 12p12 were found to be associated with HS. Both SNPs were involved in the expression of the ATP-binding cassette, subfamily C, member 9 gene (ABCC9), which encodes a “metabolic sensor” protein in astrocytes. rs73069071 can also alter the expression of the SLCO1C1 protein, which transports thyroid hormone from blood into astrocytes, and also implicated in HS pathogenesis where increased levels of T3 were found in the postmortem spinal fluid analysis of patients with HS (Nelson et al., 2016). mtDNA mutations involving single or multiple deletions were detected in the hippocampus of some mTLE patients (Yang et al., 2020). However, in our study, there was no evidence of TH as a cause of hippocampal sclerosis-associated epilepsy, which may be due to confounding factors related to hippocampal sclerosis and hippocampal sclerosis-related epilepsy.

In common genetic generalized epilepsies (GGEs), which include idiopathic generalized epilepsies (IGEs), the characteristic feature is generalized seizures, particularly absence seizures, that occur in young patients such as children and adolescents. Copy number variants (CNVs), such as 15q13.3, 15q11.2, and 16p13.11, have been identified in GGE (Mullen et al., 2018). These seizures may or may not be accompanied by automatisms or other motor symptoms. The electroencephalogram (EEG) of IGE patients typically shows bilateral symmetric 2.5–5.5 Hz spike-wave discharges (Hirsch et al., 2022). Specific subtypes within this category include CAE and JAE (Hirsch et al., 2022). With the advancement of genetic technologies, research into the etiology of these epilepsies has been continuously explored. Aberrant gene activity, such as calcium channel genes (CACNA1H, CACNG3), chloride channel genes (CLCN2), and GABA receptor genes (GABRG2, GABRA1, GABRB3, GABAB1, GABAB2), was highly associated with CAE (Thakran et al., 2020). The exact pathogenic mechanisms of JAE remain to be determined. A calcium voltage-gated channel gene (CACNB4), glutamate ionotropic receptor genes (GABRA1, GRIK1) and a calcium homeostasis-related gene (EFHC1) are considered to be associated with JAE (Sander et al., 1997; Thakran et al., 2020). Our study did not support a pathogenic role for thyroid function in either of these two types of epilepsy.

Basic experiments conducted on mammalian models have also provided in-depth analysis of the relationship between thyroid hormones and the development and differentiation of CNS progenitor cells (Rogister et al., 1999). For instance, by examining specific markers such as doublecortin (DCX), which is indicative of adult neurogenesis, researchers have gained insights into the biological defects caused by hypothyroidism, leading to structural and functional impairments in the hippocampus (Gong et al., 2010). Hypothyroidism affects hippocampal plasticity in rats by increasing cyclooxygenase-2 (COX-2) expression and proinflammatory cytokine levels (Nam et al., 2018). Similar regulatory mechanisms have been observed in various *in vivo* and *in vitro* studies. Both young mice and adult mice were tested because of the different physiologies in each life stage; for example, fetal mice rely on placental transfer of thyroid hormones due to their inability to synthesize them. Kapoor et al. (2012) reported that T3 can increase the number of DCX (+) cells and accelerate the differentiation of hippocampal neurons in adult rodents through its influence on the transcription factors Type2b and Type3. Similar studies have been reported on sonic hedgehog (Shh), which is thought to be a key factor involved in the development of neurons and oligodendrocyte precursor cells during adulthood (Ostasov et al., 2020). Desouza et al. (2011) found that thyroid hormones in adult mice regulate the expression of Shh, leading to a series of downstream signaling pathways involving membrane-associated receptors, smoothened (Smo), and patched (Ptc, Ptch1). Decreased expression of Shh is observed in hypothyroidism, while increased expression is observed in hyperthyroidism. Additionally, thyroid hormones selectively downregulate the expression of Smo mRNA in the dentate gyrus of the hippocampus, while long-term T3 intake upregulates the expression of Shh in the neocortex and dentate gyrus, further promoting the maturation of structures such as the hippocampus and neocortex. In addition, the loss or reduction in the number of inhibitory interneurons may be associated with hypothyroidism or TH transporter deficiency (Mayerl et al., 2022). Notably, structural damage-induced epilepsy resulting from other etiological factors, such as metabolic and immune factors, may not manifest as macroscopic changes in brain tissue structure. Therefore, the coexistence of etiological factors can increase the diagnostic and therapeutic challenges of the disease (Steriade et al., 2020). While basic research has indicated a close association between thyroid hormones and the development of the nervous system, our study did not support thyroid dysfunction as a causative factor for various types of epilepsy. Epilepsy cannot be solely attributed to abnormalities in the development of the nervous system.

In the latest etiological classification of epilepsy (Scheffer et al., 2017), systemic autoimmune diseases can cause damage to nerve cells by corresponding autoimmune antibodies directly or indirectly binding to ion channels and synaptic proteins on the neuronal cell membrane, such as glutamic acid decarboxylase 56 (GAD-56) and N-methyl-D-aspartate (NMDA) receptors (Armangue et al., 2013; Gresa-Arribas et al., 2015). Following the pathological immune response, core symptoms resembling infectious encephalitis can occur, including neurological deficits, behavioral abnormalities, seizures, and convulsion. As early as 1966, steroid-responsive encephalopathy associated with autoimmune thyroiditis (SREAT), also known as Hashimoto encephalopathy (HE), was proposed as a condition related to Hashimoto thyroiditis and other autoimmune thyroiditis diseases, such as Graves’ disease. It is considered the main cause of primary hypothyroidism in the pediatric population (McLeod and Cooper, 2012; Laurent et al., 2016). Patients commonly present with abnormal changes in EEG and epileptic seizures, which lack specificity. Previous reports have described a decrease in electrical baseline activity (Ercoli et al., 2019) and characteristic generalized rhythmic delta waves in HE with anti-NMDAR, with or without superimposed beta waves (extreme delta brush) (Schmitt et al., 2012). However, the localization of epileptic activity does not fully match the lesions seen on imaging, suggesting that autoantibodies may lead to widespread functional changes. This emphasizes that the microstructural alterations in HE may not be clearly visible on MRI. However, there is still debate regarding the causal relationship between the two and whether the progression and remission of HE parallel changes in thyroid function, rather than other causes of neurological changes. Our study did not find a clear causal relationship between AITDs and various types of epilepsy, warranting further investigation in the future.

Studies have elucidated the impact of thyroid hormones on the development and differentiation of CNS progenitor cells, providing insights into the biological defects caused by hypothyroidism and their association with hippocampal structural and functional impairments. Despite achieving significant research progress through various genetic study approaches, the involvement of multiple genetic variations and environmental factors in disease development cannot be fully excluded during experimental processes. This heterogeneity of contributing factors results in diverse research outcomes. Therefore, precise genetic testing for the etiology of these idiopathic epilepsies and subsequent accurate diagnosis play a crucial role in diagnosis, treatment, and disease management (Weber et al., 2017).

In recent years, there has been a growing focus on understanding epilepsy as a network disease (Royer et al., 2022). Topology and graph theory are essential theoretical foundations for brain network research. Using results from examinations such as structural and functional MRI, diffusion tensor imaging, magnetoencephalography, and electroencephalography, it is possible to calculate and analyze the brain network status of epilepsy patients (Bullmore and Sporns, 2009). Currently, invasive monitoring such as electrocorticogram (ECoG) and stereotactic electroencephalography (SEEG) play a more important role in brain network analysis and helps in the accurate localization of epileptic foci (Burns et al., 2014; Goodale et al., 2020). Various studies have highlighted distinctive features of reduced integration in epileptic brain structural networks compared to those of healthy individuals (Slinger et al., 2022). Insular epilepsy involves extensive network interactions among neocortical, subcortical, and brainstem structures (Zhao et al., 2020). Notably, individuals with epilepsy demonstrated diminished global and local brain network properties through diffusion tensor imaging (DTI) analysis. And the white matter fiber connectivity in crucial brain regions was also reduced (Wang et al., 2023). Furthermore, evidence suggests that thyroid disorder has an impact on global brain connectivity. Anti-thyroid treatment can increase functional connectivity in the regions of the left fronto-parietal network, right fronto-parietal network, and default mode network (DMN) (Kumar et al., 2022). Treatment with the thyroid hormone analog 3,3′,5-triiodothyronine acetic acid (TRIAC) has been reported to ameliorate white matter loss and brain network dysfunction associated with thyroid hormone transporter deficiency (Reinwald et al., 2022). While the aforementioned previous studies suggest that thyroid hormones may influence the occurrence of epilepsy by modulating brain networks, our study did not identify a causal relationship between thyroid hormones and epilepsy. Further in-depth research may be needed in the future to explore the potential mechanism.

Previous studies have analyzed the potential relationship between thyroid function and epilepsy (Tamijani et al., 2015; Sawicka-Gutaj et al., 2022). We aim to further investigate the potential causal relationships within this context. However, after conducting a thorough analysis using MR methods, we did not find significant causal relationships therein. Further basic medical research would be helpful to provide in-depth understanding of our study. This study has several limitations. First, the populations included in the screening of SNP loci associated with thyroid function and epilepsy primarily consisted of individuals of European ancestry. Further validation is needed in other ethnicities to determine if similar results can be observed. Additionally, when different methods were used to validate certain results, inconsistencies were observed, particularly in the study on AITDs and generalized epilepsy (all documented cases). This may be attributed to the inclusion of various diseases within AITDs, such as Graves’ disease and Hashimoto’s thyroiditis, which were not individually studied. In the future, it will be necessary to rely on new large-scale database data that categorize different types of AITD-related diseases for better MR analysis.

## 5 Conclusion

Our MR analysis indicates that there is no causal relationship between thyroid disorders and various types of epilepsy. Future research simultaneously involving these two types of diseases should proceed with greater caution to avoid potential confounding factors that may impact study outcomes.

## Data availability statement

The original contributions presented in this study are included in this article/Supplementary material, further inquiries can be directed to the corresponding authors.

## Author contributions

DL: Formal Analysis, Writing – original draft, Conceptualization, Visualization. YW: Writing – original draft, Formal Analysis, Validation. YY: Writing – review & editing, Validation. HZ: Writing – review & editing, Methodology. XF: Writing – review & editing, Investigation, Resources. SC: Writing – review & editing, Methodology, Resources. PW: Writing – review & editing, Conceptualization, Methodology. YS: Funding acquisition, Supervision, Writing – review & editing. GZ: Funding acquisition, Supervision, Writing – review & editing, Conceptualization.

## Footnotes

1
https://transfer.sysepi.medizin.uni-greifswald.de/thyroidomics/

2
https://www.ilae.org/

3
https://www.mrbase.org/

4
https://www.ebi.ac.uk/gwas/

5
http://www.phenoscanner.medschl.cam.ac.uk/

6
https://pubmed.ncbi.nlm.nih.gov/

## Supplementary material

Supplementary Table 1 Thyroid function SNPs and Mendelian randomization (MR) results.
